# Supplementary material for: Enhanced Representation-Based Sampling for the Efficient Generation of Data Sets for Machine-Learned Interatomic Potentials
Source: J Chem Theory Comput. 2026 Feb 2;22(4):1947–57. doi: 10.1021/acs.jctc.5c01767 (PMC12937107; doi:10.1021/acs.jctc.5c01767)
Supplement: Supplementary file 1 [file ct5c01767_si_001.pdf]

Enhanced Representation-Based Sampling for the Efficient Generation of Datasets for Machine-Learned Interatomic Potentials

Moritz R. Schäfer and Johannes Kästner*

Institute for Theoretical Chemistry, University of Stuttgart, Pfaffenwaldring 55, 70569 Stuttgart, Germany

E-mail: kaestner@theochem.uni-stuttgart.de

Supporting Information Available

S1 Model Hyperparameters

All hyperparameters used to train the models of each experiment are listed in Table S1. The Huber loss function used for the alanine dipeptide experiment is given by

$$\mathcal{L}_\delta(\boldsymbol{\theta}) = \sum_{k=1}^{N_{\text{train}}} \sum_{i}^{N_{\text{atoms}}^{(k)}} \frac{1}{3N_{\text{atoms}}^{(k)}} \begin{cases} \frac{1}{2}(\boldsymbol{F}_{i,k}^{\text{ref}} - \boldsymbol{F}_i(S_k, \boldsymbol{\theta}))^2, & \text{if } |\boldsymbol{F}_{i,k}^{\text{ref}} - \boldsymbol{F}_i(S_k, \boldsymbol{\theta})| \leq \delta \\ \delta\left(|\boldsymbol{F}_{i,k}^{\text{ref}} - \boldsymbol{F}_i(S_k, \boldsymbol{\theta})| - \frac{1}{2}\delta\right), & \text{otherwise} \end{cases} \tag{S1}$$

In total, the number of trainable parameters was 32929 for the Alanine Dipeptide, 90244 for water, and 92548 $BMIM^{+}BF_4{}^{-}$ experiments. The difference in parameter counts for the last two experiments arises from the different number of elements in the systems, which results in a different number of elemental embedding parameters.

S2 Scaling

The method by Yoo et al. constructs per-atom bias potentials based on Behler-Parinello descriptors and a metadynamics-like functional form of the bias. The use of uncompressed per-atom descriptors significantly increases the memory requirements for storing the reference descriptors. Further, the calculation of E_{bias} requires comparison of each atom's descriptor with all reference descriptors of the same element. This introduces an additional factor to the cost calculation of the bias potential of N_{atoms}. ERBS uses diagonal kernels; the approach of Yoo et al.[1] constructs an adaptive covariance matrix from descriptor Jacobians according to:

$$\Sigma_{jk}(\mathbf{G}) = \sigma^2 \sum_{i=1}^{N_{\text{at}}} \sum_{\alpha=x,y,z} \frac{\partial G_j}{\partial R_{i,\alpha}} \frac{\partial G_k}{\partial R_{i,\alpha}} + \varepsilon \delta_{jk}, \tag{S2}$$

Table S1: GMNN training hyperparameters used throughout the experiments of Alanine Dipeptide, Water and $BMIM^+BF_4{}^-$

**Hyperparameter**	**Alanine Dipeptide**	**Water, $BMIM^+BF_4{}^-$**
Training		
Epochs	10 000	10 000
Batch size	8	1
Gradient clipping	10.0	10.0
Model		
Basis function	Bessel, $n = 16$, $r_{\text{max}} = 5.0$	Bessel, $n = 16$, $r_{\text{max}} = 5.5$
Radial functions	5	6
NN layers	64, 64	128, 64
Ensemble	—	Shallow (16 members)
Optimizer		
Name	AdamW	AdamW
Embedding LR	0.001	0.0001
NN LR	0.001	0.0001
Scale LR	0.0005	0.0001
Shift LR	0.0005	0.0001
Weight decay	10^{-5}	$2 \cdot 10^{-4}$
Schedule		
Name	Cyclic cosine	Cyclic cosine
Period	50	50
Decay factor	0.95	0.96
Loss functions		
Energy loss	Huber ($\delta = 0.5$), weight=1.0	NLL, weight=1.0
Force loss	Huber ($\delta = 0.1$), weight=2.0	NLL, weight=2.0

where σ is a hyperparameter controlling the scale of the kernel, and ϵ is a regularization constant added to the diagonal to ensure numerical stability. The covariance matrix has a dimensionality of $N_{\text{feat}} \times N_{\text{feat}}$, where N_{feat} is the number of descriptor components per configuration. Its inverse needs to be stored along with the reference descriptors or recomputed on the fly, adding to either the memory or computational cost.

Additionally, we find that, at least for the GM descriptor, this matrix can be severely ill-conditioned, such that its inverse is dominated by the choice of ϵ, effectively suppressing the geometry-dependent structure of the covariance. One possible cause for the ill-conditioning

is that the GM descriptor constructs many body features via outer products, leading to high correlation of the features. The Behler-Parinello descriptor, on the other hand, has separate parts for two and three-body interactions of element pairs and triplets, reducing the correlation.

S3 Ramachandran Space Coverage

To further analyze the exploration behavior of the ERBS method in comparison to high-temperature molecular dynamics, we investigate the Ramachandran space coverage visually. Figure S1 displays the coverage plots for the MD 1200 K, ERBS A, ERBS B trajectories, and one that led to the dissociation of the molecule. The parameter choice for the dissociated simulation was $\Delta E = 10$ eV, $\sigma = 0.1$, and $k = 2$. We find that the MD simulation at 1200 K does not sample the free energy minimum in the lower right quadrant, while both ERBS simulations do. The good description of the minimum location and high errors on the right-hand side of the FES of the MD 1200 K model can be explained in this way. The broken simulation achieves a high coverage, although this is merely due to the free rotation of fragments.

S4 Radial Distribution Functions of Water

We further investigate the capability of the active learned MLIP to reproduce structural properties of liquid water. Specifically, we calculate the oxygen-oxygen radial distribution functions (RDFs). As shown in Figure S2, the RDFs for all models are in good agreement and visually indistinguishable, accurately reproducing the location and intensity of both the first and second hydration shells.

We do not observe an underestimation of the hydration shells that would explain an overestimated diffusion coefficient. The radial distribution function defines the effective 2-body potential of mean force (PMF), $W(r) = -k_{\mathrm{B}}T \log g(r)$. However, $W(r)$ is a free energy

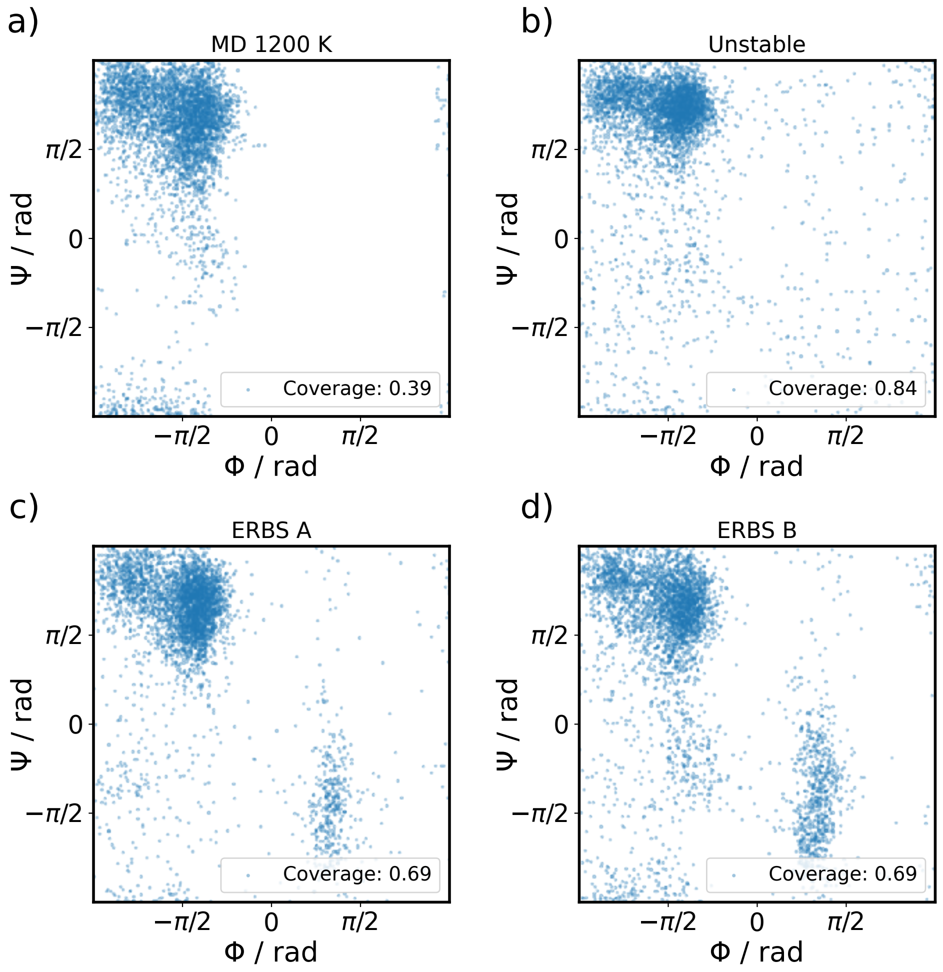

Figure S1: Ramachandran space coverage of the a) MD 1200K, b) $\Delta E = 10$ eV, $\sigma = 0.1$, and $k = 2$, c) ERBS A, and d) ERBS B.

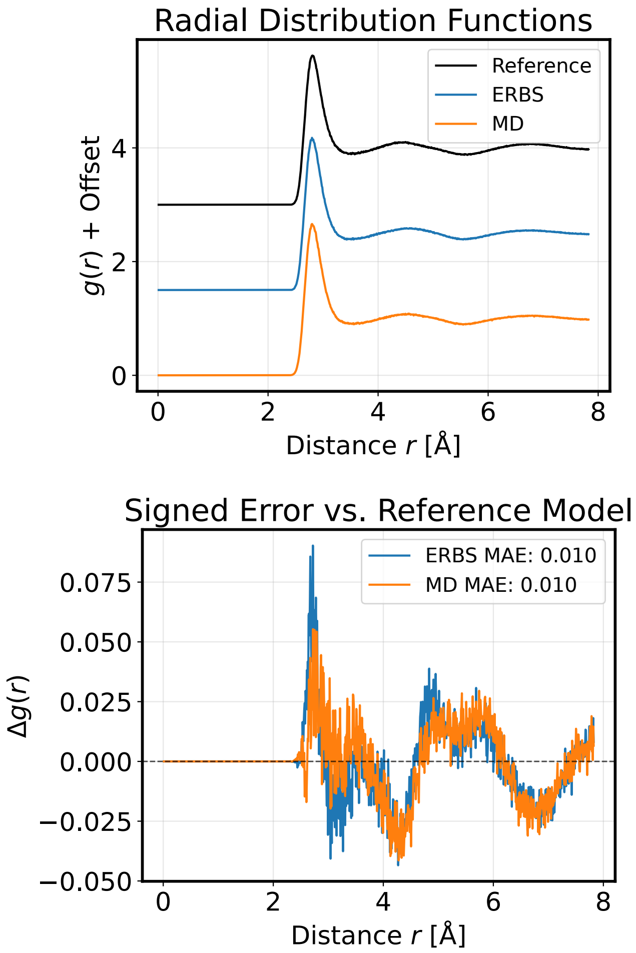

Figure S2: Oxygen-oxygen radial distribution functions of the reference model and the MLIPs produced by active learning on MD and ERBS data. The right panel shows the signed error of the active learned models with respect to the reference.

surface resulting from integrating out all other degrees of freedom, including higher body order terms.

Diffusion, however, is a dynamical process governed by the underlying high-dimensional potential energy surface, not the projected PMF. A transition event often requires a specific many-body rearrangement, such as a rotation around a bond that constitutes a high-energy barrier on the true PES.

The fact that the MD-trained model reproduces the RDF, and thus the potential of mean force, but overestimates diffusion implies it has learned a 'flattened' PES. It captures the pairwise energetics correctly but lacks the explicit many-body repulsions that create the true friction and transition barriers in the liquid.

S5 Calibration Metrics

Assessing the quality of uncertainty estimates is crucial in the active learning context, both for terminating sampling trajectories and for the appropriate biasing by UDD. Figure S3 displays the scatter plots for predicted uncertainties compared to empirical errors for energy and force uncertainties for shallow ensembles trained with MSE and NLL losses. While the validation errors are comparable for both cases, the calibration of uncertainty estimates is significantly improved by the use of the probabilistic loss function.

S6 Force Decomposition

In molecular liquids, the forces on each atom can be analytically decomposed into vibrational, translational, and rotational forces.[2] Figure S4 shows the prediction errors of the model trained on $BMIM^+BF_4^-$ for the decomposed forces. The separation in magnitude between inter- and intramolecular forces is evident. While the MAE for the intermolecular forces is fairly small, so are the true forces. The mean absolute force components of the translational and rotational are $20.6\,\mathrm{meV\,Å^{-1}}$ and $19.5\,\mathrm{meV\,Å^{-1}}$ respectively, resulting in large relative

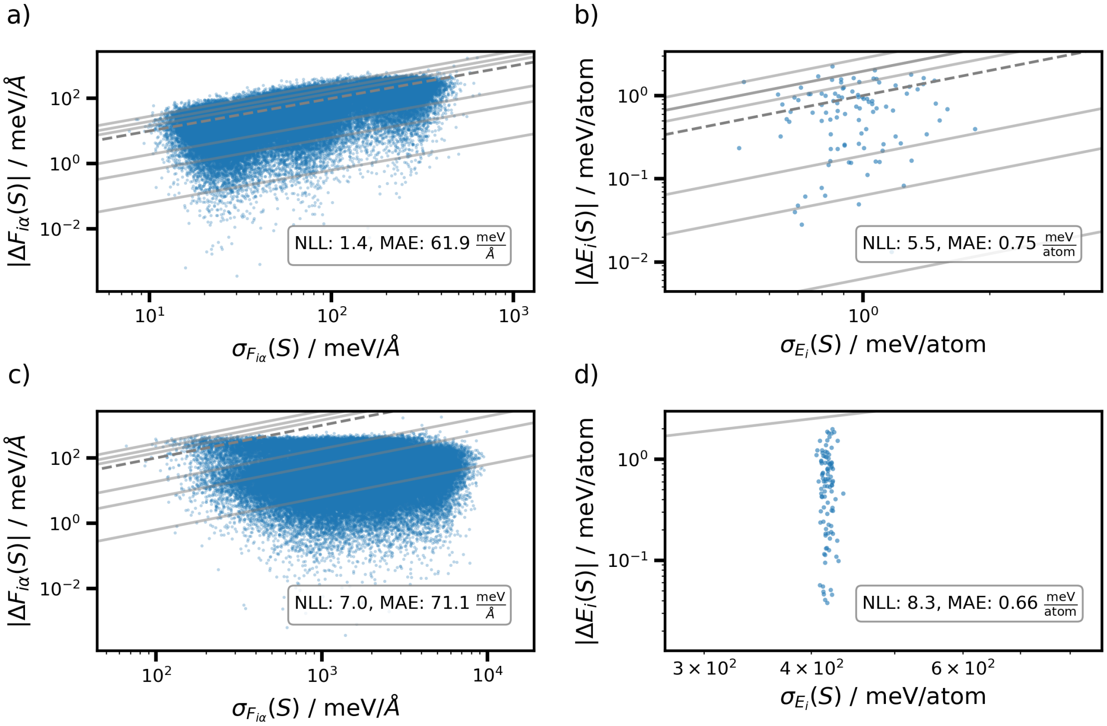

Figure S3: Predicted-empirical error scatter plot for energies and forces of the $BMIM^+BF_4^-$ validation dataset. Panels a) and b) show the performance of the shallow ensemble trained with an NLL loss, c) and d) that of the shallow ensemble trained with an MSE loss.

errors.

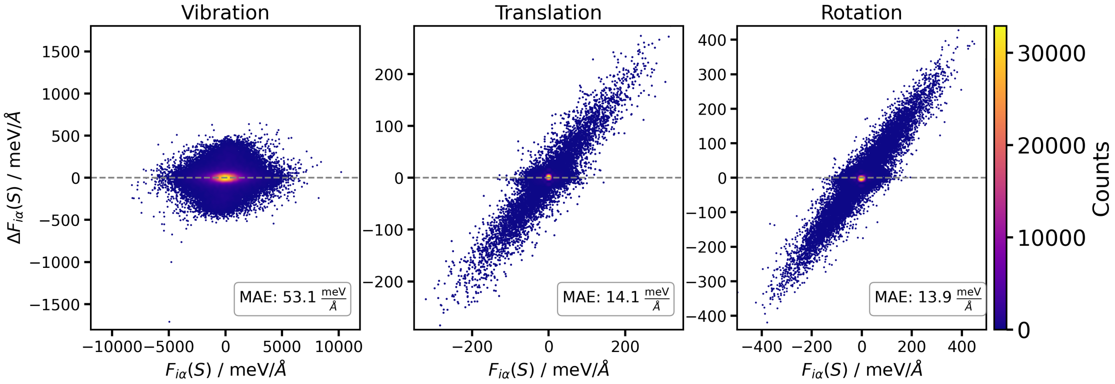

Figure S4: Error of predicted compared to true force components for the $BMIM^+BF_4{}^-$ validation dataset.

S7 Test Set Error Analysis

To evaluate predictive quality, we present density-colored parity plots of energy and force errors for key models. For the alanine dipeptide, where cross-validation results are detailed in the main text, Figure S5 shows the self-validation performance. As the reference data is derived from a classical force field, all models fit energies and forces well below chemical accuracy.

For the water experiment, we compare the final MD and ERBS active learning models against the model trained on the literature dataset. While the MD and ERBS models achieve similar high accuracy, the literature-trained model exhibits consistently higher errors. This is attributed to the broader diversity of the literature dataset, which includes path-integral MD and constant-pressure simulations.

Finally, for $BMIM^+BF_4{}^-$, we analyze the shallow ensemble trained on the literature dataset. It achieves improved energy metrics compared to the original publication (MAE: 5.5 meV atom^{-1}), though force metrics are slightly higher (reference MAE: 48 meV Å^{-1}).

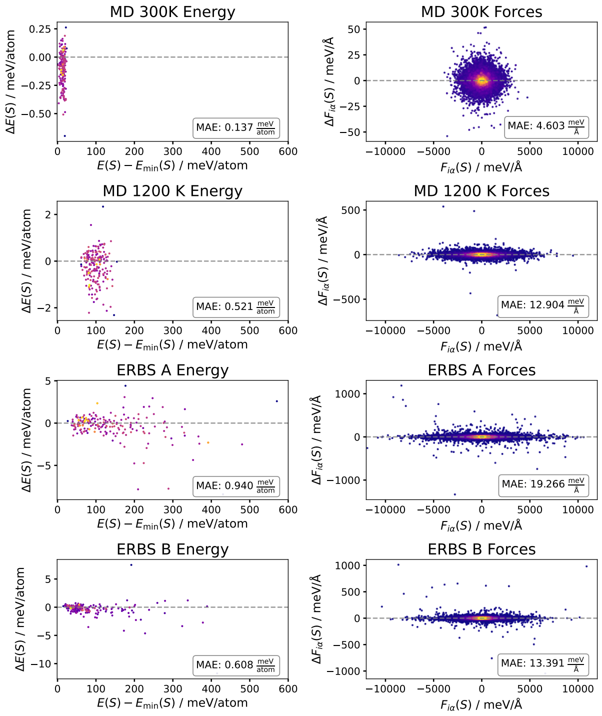

Figure S5: Energy and force prediction errors of the MD 300 K, MD 1200 K, ERBS A and ERBS B on their respective validation sets.

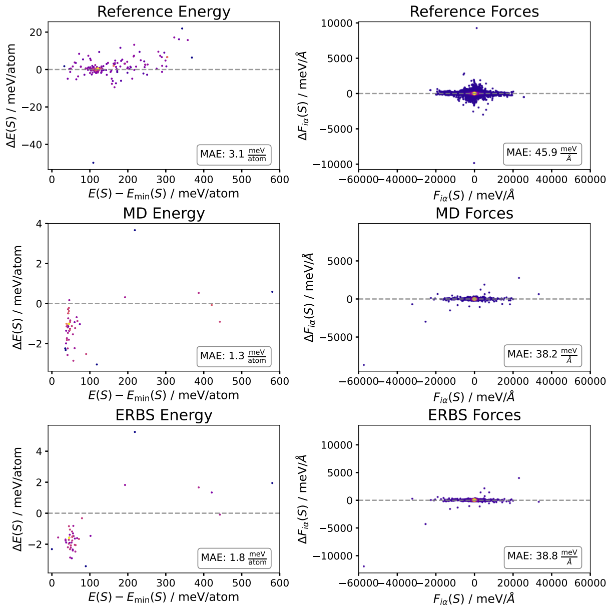

Figure S6: Energy and force prediction errors of the reference model and the active learned models trained on MD and ERBS data.

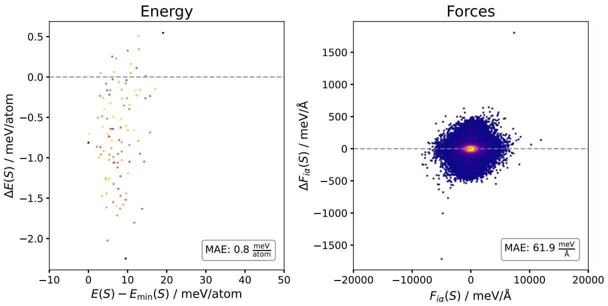

Figure S7: Energy and force prediction errors of the model trained on the $BMIM^+BF_4^-$ literature dataset.

References

(1) Yoo, D.; Jung, J.; Jeong, W.; Han, S. Metadynamics Sampling in Atomic Environment Space for Collecting Training Data for Machine Learning Potentials. *npj Computational Materials* **2021**, *7*, 1–9.

(2) Magdău, I.-B.; Arismendi-Arrieta, D. J.; Smith, H. E.; Grey, C. P.; Hermansson, K.; Csányi, G. Machine Learning Force Fields for Molecular Liquids: Ethylene Carbonate/Ethyl Methyl Carbonate Binary Solvent. *npj Computational Materials* **2023**, *9*, 1–15.
